# Differential roles of trans-phosphorylated EGFR, HER2, HER3, and RET as heterodimerisation partners of MET in lung cancer with *MET* amplification

**DOI:** 10.1038/bjc.2011.322

**Published:** 2011-08-16

**Authors:** J Tanizaki, I Okamoto, K Sakai, K Nakagawa

**Affiliations:** 1 Department of Medical Oncology, Kinki University Faculty of Medicine, 377-2 Ohno-higashi, Osaka-Sayama, Osaka 589-8511, Japan; 2 Genome Biology, Kinki University Faculty of Medicine, 377-2 Ohno-higashi, Osaka-Sayama, Osaka 589-8511, Japan

**Keywords:** *MET* amplification, trans-phosphorylation, heterodimerisation, lung cancer

## Abstract

**Background:**

MET is a receptor tyrosine kinase (RTK) whose gene is amplified in various tumour types. We investigated the roles and mechanisms of RTK heterodimerisation in lung cancer with *MET* amplification.

**Methods:**

With the use of an RTK array, we identified phosphorylated RTKs in lung cancer cells with *MET* amplification. We examined the roles and mechanisms of action of these RTKs with immunoprecipitation, annexin V binding, and cell migration assays.

**Results:**

We identified epidermal growth factor receptor (EGFR), human EGFR (HER)2, HER3, and RET in addition to MET as highly phosphorylated RTKs in lung cancer cells with *MET* amplification. Immunoprecipitation revealed that EGFR, HER2, HER3, and RET each formed a heterodimer exclusively with MET and that these associations were markedly reduced in extent by treatment with a MET kinase inhibitor. RNA interference-mediated depletion of EGFR, HER2, or HER3 induced apoptosis in association with inhibition of AKT and ERK signalling pathways, whereas depletion of HER2 or RET inhibited both cell migration and STAT3 signalling.

**Conclusion:**

Our data suggest that heterodimers of MET with EGFR, HER2, HER3, or RET have differential roles in tumour development, and they provide new insight into the function of trans-phosphorylated RTKs as heterodimerisation partners of MET in lung cancer with *MET* amplification.

The proto-oncogene *MET* encodes a receptor tyrosine kinase (RTK). Amplification of *MET* occurs in a subset of solid tumour types, with aberrant MET signalling having been implicated in cell proliferation and survival as well as in cell migration and invasiveness (Trusolino and Comoglio, 2002; Birchmeier *et al*, 2003). Tumours with *MET* amplification are highly dependent on MET signalling for their progression, and several small-molecule inhibitors of MET have been developed and found to manifest marked antitumour efficacy both *in vitro* and *in vivo* (Smolen *et al*, 2006; Lutterbach *et al*, 2007; Okamoto *et al*, 2010). Such inhibitors are now undergoing clinical trials in humans.

Lung cancer is the leading cause of cancer mortality worldwide. Molecularly targeted agents, such as epidermal growth factor receptor (EGFR)–tyrosine kinase inhibitors (TKIs), have been found to provide substantial clinical benefit in lung cancer patients (Asahina *et al*, 2006; Sequist *et al*, 2008; Kwak *et al*, 2010), and MET is considered a molecular target of potential relevance to lung cancer. Amplification of *MET* occurs in ∼4% of lung cancer patients, with activation of MET signalling being associated with advanced cancer stage and shorter patient survival (Masuya *et al*, 2004; Zhao *et al*, 2005; Nakamura *et al*, 2007). MET–TKIs are currently undergoing early-phase clinical testing in lung cancer patients. Optimisation of the clinical efficacy of such drugs will require further characterisation of MET signal transduction in tumours with *MET* amplification. Although MET has been found to associate with other RTKs (Jo *et al*, 2000; Engelman *et al*, 2007; Lai *et al*, 2009; Mueller *et al*, 2010), the underlying mechanisms and regulation of such association in lung cancer with *MET* amplification remain largely unclear.

We have now shown that several RTKs, including EGFR, human EGFR (HER)2, HER3, and RET in addition to MET, are highly phosphorylated in *MET* amplification-positive lung cancer cells, and that these RTKs form heterodimers with MET. Such heterodimerisation results in trans-phosphorylation of these RTKs in a manner dependent on the kinase activity of MET. We further investigated the functional roles of these RTKs in lung cancer cells with *MET* amplification.

## Materials and methods

### Cell culture and reagents

The human non-small cell lung cancer (NSCLC) cell lines H1993, PC9, and HCC827, the human breast cancer cell lines SK-BR3 and BT-474, and the human medullary thyroid carcinoma cell line TT were obtained from American Type Culture Collection (Manassas, VA, USA). The human NSCLC cell line EBC-1 was obtained from the Health Science Research Resources Bank (Tokyo, Japan). SK-BR3 cells were cultured in McCoy's medium (Invitrogen, Carlsbad, CA, USA) supplemented with 10% fetal bovine serum (FBS); BT-474 cells in Dulbecco's modified Eagle's medium (Invitrogen) supplemented with 10% FBS; TT cells in Ham's F-12 medium (Sigma, St Louis, MO, USA) supplemented with 10% FBS; and other cells in RPMI 1640 medium (Sigma) supplemented with 10% FBS. All cell lines were maintained under a humidified atmosphere of 5% CO_2_ at 37 °C. PHA-665752 was obtained from Tocris Bioscience (Bristol, UK), gefitinib was from Kemprotec (Middlesbrough, UK), lapatinib was from Sequoia Research Products (Pangbourne, UK), and vandetanib was from ShangHai Biochempartner (Shanghai, China).

### RTK array analysis

Tyrosine-phosphorylated RTKs were detected with the use of Array 001 (R&D Systems, Minneapolis, MN, USA), which contains capture antibodies to 42 RTKs in duplicate wells. Cell lysates were incubated overnight at 4 °C with the array in the provided buffer. Target RTKs are captured by the respective capture antibodies, and tyrosine-phosphorylated RTKs are subsequently detected with horseradish peroxidase-conjugated antibodies to phosphotyrosine.

### Immunoblot analysis

Cells were washed twice with ice-cold phosphate-buffered saline (PBS) and then lysed in a solution containing 20 mM Tris-HCl (pH 7.5), 150 mM NaCl, 1 mM EDTA, 1% Triton X-100, 2.5 mM sodium pyrophosphate, 1 mM phenylmethylsulfonyl fluoride, and leupeptin (1 *μ*g ml^–1^). The protein concentration of cell lysates was determined with the use of a BCA protein assay kit (Thermo Fischer Scientific, Waltham, MA, USA), and equal amounts of lysate protein were subjected to SDS–polyacrylamide gel electrophoresis on a 7.5 or 12% gel (Bio-Rad, Hercules, CA, USA). The separated proteins were transferred to a nitrocellulose membrane, which was then incubated with Blocking One solution (Nacalai Tesque, Kyoto, Japan) for 20 min at room temperature before incubation overnight at 4 °C with primary antibodies. Rabbit polyclonal antibodies to phosphorylated human MET (pY1234/pY1235), to phosphorylated EGFR (pY1068), to phosphorylated HER2 (pY1221), to phosphorylated HER3 (pY1289), to phosphorylated RET (pY905), to AKT, to phosphorylated AKT, to signal transducer and activator of transcription 3 (STAT3), to phosphorylated STAT3, and to poly(ADP-ribose) polymerase (PARP) were obtained from Cell Signaling Technology (Danvers, MA, USA); those to HER3, to RET, to extracellular signal-regulated kinase (ERK), and to phosphorylated ERK were from Santa Cruz Biotechnology (Santa Cruz, CA, USA); those to MET were from Zymed (South San Francisco, CA, USA); those to HER2 were from Millipore (Billerica, MA, USA); and those to *β*-actin were from Sigma. Mouse monoclonal antibodies to EGFR were obtained from Invitrogen. All antibodies were used at a 1 : 1000 dilution, with the exception of those to *β*-actin (1 : 200) and to EGFR (1 : 100). After incubation with primary antibodies, the membrane was washed with PBS containing 0.05% Tween 20 before incubation for 1 h at room temperature with horseradish peroxidase-conjugated goat antibodies to rabbit or mouse immunoglobulin (Ig) G (Sigma). Immune complexes were finally detected with chemiluminescence reagents (GE Healthcare, Little Chalfont, UK).

### Immunoprecipitation

Total cell lysates (800 *μ*g of protein) were incubated overnight at 4 °C either with antibodies to MET (Cell Signaling), to EGFR (Cell Signaling), to HER2 (Cell Signaling), to HER3 (Santa Cruz Biotechnology), or to RET (Santa Cruz Biotechnology) or with control IgG (Cell Signaling). Immune complexes were then precipitated by further incubation for 1 h at 4 °C with a suspension of protein G- and protein A-conjugated agarose (Calbiochem, Darmstadt, Germany). Immunoprecipitates were isolated, washed, resolved by SDS–polyacrylamide gel electrophoresis on a 7.5% gel, and subjected to immunoblot analysis as described above.

### Gene silencing

Cells were plated at 50–60% confluence in six-well plates or 25-cm^2^ flasks and then incubated for 24 h before transient transfection for the indicated times with small interfering RNAs (siRNAs) mixed with the Lipofectamine reagent (Invitrogen). The siRNAs specific for MET (MET-1, 5′-ACAAGAUCGUCAACAAAAA-3′ MET-2, 5′-CUACAGAAAUGGUUUCAAA-3′), EGFR (EGFR-1, 5′-GAAAUAUGUACUACGAAA-3′ EGFR-2, 5′-GGAACUGGAUAUUCUGAAA-3′), HER2 (HER2-1, 5′-CCAUUGAUGUCUACAUGAU-3′ HER2-2, 5′-AGGACAUCUUCCACAAGAA-3′), HER3 (HER3-1, 5′-UCUUCGUCAUGUUGAACUA-3′ HER3-2, 5′-CCUCCUUGAUGAUGACCCA-3′), or RET (RET-1, 5′-GGGAUGCUUACUGGGAGAA-3′ RET-2, 5′-CCACCCACAUGUCAUCAAA-3′) mRNAs as well as nonspecific (control) siRNAs were obtained from Nippon EGT (Toyama, Japan). The data presented were obtained with MET-1, EGFR-1, HER2-1, HER3-1, and RET-1 siRNAs, but similar results were obtained with MET-2, EGFR-2, HER2-2, HER3-2, and RET-2.

### Cell proliferation assay

Cells were transferred to 96-well flat-bottomed plates and cultured for 24 h before exposure to various agents or transfection with siRNAs for 72 h. TetraColor One (5 mM tetrazolium monosodium salt and 0.2 mM 1-methoxy-5-methyl phenazinium methylsulphate; Seikagaku, Tokyo, Japan) was then added to each well, and the cells were incubated for 3 h at 37 °C before measurement of absorbance at 490 nm with a Multiskan Spectrum instrument (Thermo Labsystems, Boston, MA, USA). Absorbance values were expressed as a percentage of that for control cells.

### Annexin V-binding assay

Binding of annexin V to cells was measured with the use of an annexin-V-FLUOS Staining Kit (Roche, Basel, Switzerland). Cells were harvested by exposure to trypsin-EDTA, washed with PBS, and centrifuged at 200 × **g** for 5 min. The cell pellets were resuspended in 100 *μ*l of annexin-V-FLUOS labelling solution, incubated for 10–15 min at 15–25 °C, and then analysed for fluorescence with a flow cytometer (FACSCalibur, Becton Dickinson, Franklin Lakes, NJ, USA) and Cell Quest software (Becton Dickinson).

### Cell migration assay

Cells were transfected with siRNAs specific for MET, EGFR HER2, HER3, or RET mRNAs for 24 h and were then transferred in serum-free medium to cell culture inserts (pore size, 8 *μ*m; BD Falcon, Franklin Lakes, NJ, USA) of a transwell apparatus. Complete medium (containing 10% FBS) served as the chemoattractant in the lower chamber. After culture for 24 h, cells that had migrated through the membrane pores were fixed and stained. Cell migration was quantified by counting the numbers of cells in five randomly chosen microscopic fields of view (magnification, × 400) per membrane, and the mean value was then expressed relative to that for cells transfected with a control siRNA.

## Results

### EGFR, HER2, HER3, and RET are highly phosphorylated in a manner dependent on MET kinase activity in lung cancer cell lines with *MET* amplification

**Figure 1:**
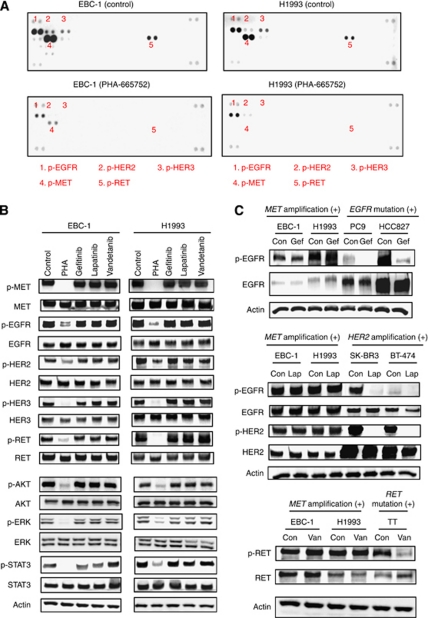
Phosphorylation of multiple RTKs in lung cancer cells with *MET* amplification. (**A**) EBC-1 and H1993 cells were deprived of serum for 24 h and then incubated for 2 h in the absence (control) or presence of PHA-665752 (500 nM). Cell lysates were prepared and incubated with an RTK array for determination of the phosphorylation status of each enzyme. Each RTK is spotted in duplicate, and the pairs of dots in each corner of the array are positive controls. The numbered pairs of dots correspond to the indicated phosphorylated (p-) RTKs. (**B**) EBC-1 and H1993 cells were deprived of serum for 24 h and then incubated for 2 h in the absence or presence of PHA-665752 (PHA, 500 nM), gefitinib (1 *μ*M), lapatinib (1 *μ*M), or vandetanib (1 *μ*M), after which cell lysates were subjected to immunoblot analysis with antibodies to phosphorylated or total forms of MET, EGFR, HER2, HER3, RET, AKT, ERK, or STAT3 or with those to *β*-actin (loading control). (**C**) The indicated cell lines were deprived of serum for 24 h and then incubated for 2 h in the absence (Con) or presence of gefitinib (Gef, 1 *μ*M), lapatinib (Lap, 1 *μ*M), or vandetanib (Van, 1 *μ*M), after which cell lysates were subjected to immunoblot analysis with antibodies to the indicated proteins.

We first examined the phosphorylation of RTKs in lung cancer cells positive for *MET* amplification. With the use of an RTK array, we found that EBC-1 and H1993, two human lung cancer cell lines with *MET* amplification, exhibited a high level of tyrosine phosphorylation of MET, EGFR, HER2, HER3, and RET under conditions of serum deprivation (Figure 1A). The MET inhibitor PHA-665752 markedly reduced the level of MET phosphorylation as well as that of EGFR, HER2, HER3, and RET phosphorylation (Figure 1A). Consistent with these results obtained with the RTK array, immunoblot analysis showed that PHA-665752 inhibited the phosphorylation of MET, EGFR, HER2, HER3, and RET as well as that of the downstream signalling molecules AKT, ERK, and STAT3 in both EBC-1 and H1993 cells (Figure 1B). To investigate whether the observed phosphorylation of these RTKs is mediated by the corresponding intrinsic kinase activities, we examined the effects of various TKIs. The EGFR–TKI gefitinib had little effect on the phosphorylation of EGFR or that of AKT, ERK, or STAT3 in EBC-1 or H1993 cells (Figure 1B), whereas it markedly inhibited the phosphorylation of these molecules in *EGFR* mutation-positive lung cancer cell lines (PC9 and HCC827) that manifest constitutive activation of the EGFR kinase (Figure 1C, data not shown). Neither lapatinib (a dual TKI of EGFR and HER2) nor vandetanib (a TKI of RET) had a substantial effect on the phosphorylation of any of the RTKs or their downstream signalling molecules in EBC-1 or H1993 cells (Figure 1B), whereas these drugs inhibited their specific target RTKs in *HER2* amplification-positive breast cancer cell lines (SK-BR3 and BT-474) or *RET* mutation-positive medullary thyroid carcinoma TT cells, respectively (Figure 1C). These data thus suggested that phosphorylation of MET, EGFR, HER2, HER3, and RET as well as that of the downstream signalling molecules is dependent on the kinase activity of MET, but is independent of that of the other RTKs, in *MET* amplification-positive lung cancer cells.

### EGFR, HER2, HER3, and RET associate with MET in lung cancer cells with *MET* amplification

**Figure 2:**
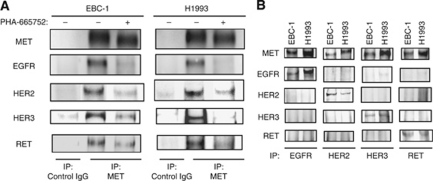
Association of MET with EGFR, HER2, HER3, and RET in lung cancer cells positive for *MET* amplification. (**A**) Serum-deprived EBC-1 and H1993 cells were incubated for 2 h in the absence or presence of PHA-665752 (500 nM), lysed, and subjected to immunoprecipitation (IP) with antibodies to MET or control IgG. The resulting precipitates were subjected to immunoblot analysis with antibodies to the indicated proteins. (**B**) Serum-deprived cells were lysed and subjected to IP with antibodies to EGFR, to HER2, to HER3, or to RET, and the resulting precipitates were subjected to immunoblot analysis with antibodies to the indicated proteins.

To investigate further the mechanism of EGFR, HER2, HER3, and RET phosphorylation in *MET* amplification-positive cells, we examined the interactions among these RTKs by immunoprecipitation analysis. MET was immunoprecipitated from EBC-1 and H1993 cell lysates, and the resulting precipitates were subjected to immunoblot analysis with antibodies to MET, to EGFR, to HER2, to HER3, and to RET. EGFR, HER2, HER3, and RET co-precipitated with MET from both cell lines, and the extent of these associations was markedly reduced by prior treatment of the cells with PHA-665752 (Figure 2A). Reciprocal immunoprecipitation analysis further revealed that MET, but not HER2, HER3, or RET, co-precipitated with EGFR and that only MET co-precipitated with HER2, HER3, or RET (Figure 2B). These data thus suggested that phosphorylation of EGFR, HER2, HER3, and RET in *MET* amplification-positive lung cancer cells is mediated through interaction of these RTKs with MET.

### Effects of TKIs on cell proliferation and apoptosis in lung cancer cells with *MET* amplification

**Figure 3:**
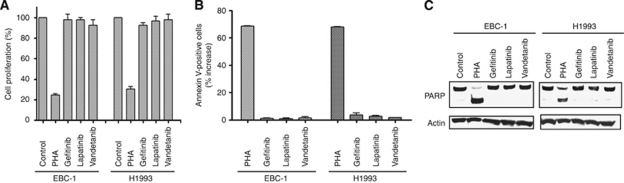
Effects of various TKIs on cell proliferation and apoptosis in lung cancer cells with *MET* amplification. (**A**) EBC-1 and H1993 cells were cultured for 72 h in complete medium with or without PHA-665752 (500 nM), gefitinib (1 *μ*M), lapatinib (1 *μ*M), or vandetanib (1 *μ*M), after which the number of viable cells was determined. Absorbance values were expressed as a percentage of that for untreated cells (control). Data are means±s.e. from three independent experiments. (**B**) Cells were incubated for 72 h as in (**A**), after which the number of apoptotic cells was determined by staining with annexin V and propidium iodide followed by flow cytometry. Data are expressed as the percentage increase in the number of annexin V-positive cells relative to the corresponding value for cells incubated without agent and are means±s.e. from three independent experiments. (**C**) Cells incubated as in (**A**) were lysed and subjected to immunoblot analysis with antibodies to PARP and to *β*-actin.

To examine the roles of MET, EGFR, HER2, HER3, and RET in lung cancer cells with *MET* amplification, we first investigated the effects of PHA-665752, gefitinib, lapatinib, and vandetanib on cell proliferation and survival. PHA-665752 inhibited the proliferation of EBC-1 and H1993 cells, whereas gefitinib, lapatinib, and vandetanib had no such effect (Figure 3A), consistent with our findings that AKT, ERK, and STAT3 signalling pathways are not dependent on the kinase activity of EGFR, HER2, or RET in these cells (Figure 1B). We further found that PHA-665752, but not gefitinib, lapatinib, or vandetanib, induced marked apoptosis in EBC-1 and H1993 cells (Figure 3B). A substantial level of PARP cleavage, a characteristic of apoptosis, was also apparent only in the cells treated with PHA-665752 (Figure 3C). These results thus suggested that only the MET–TKI induced apoptosis, resulting in inhibition of cell growth, in *MET* amplification-positive cells.

### Effects of depletion of MET, EGFR, HER2, HER3, or RET on cell proliferation, apoptosis, and migration in *MET* amplification-positive lung cancer cells

**Figure 4:**
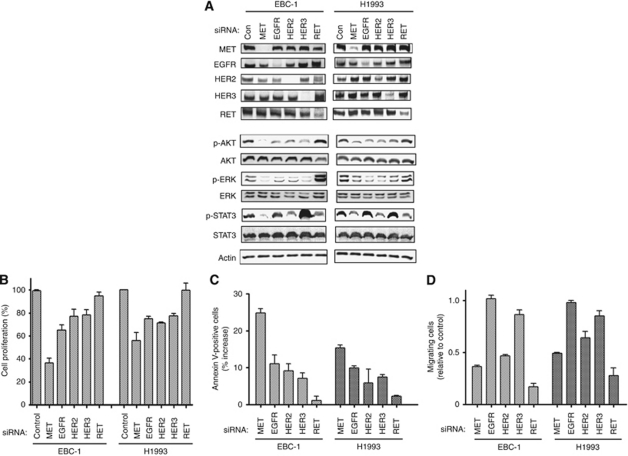
Effects of depletion of MET, EGFR, HER2, HER3, or RET on cell proliferation, apoptosis, and migration in lung cancer cells with *MET* amplification (**A**), EBC-1 and H1993 cells were transfected with MET, EGFR, HER2, HER3, RET, or nonspecific (Con) siRNAs for 72 h, after which cell lysates were prepared and subjected to immunoblot analysis with antibodies to the indicated proteins. (**B**) Cells transfected as in (**A**) were evaluated for cell proliferation. Absorbance values were expressed as a percentage of that for cells transfected with a control siRNA. (**C**) Cells transfected as in (**A**) were evaluated for the proportion of apoptotic cells. Data are expressed as the percentage increase in the number of annexin V-positive cells relative to the corresponding value for cells transfected with a control siRNA. (**D**) Cells were transfected with the indicated siRNAs for 24 h and then transferred in serum-free medium to cell culture inserts of a transwell apparatus for 24 h. The number of cells that migrated toward complete medium was counted with the use of a light microscope. Data are expressed relative to the value for cells transfected with a control siRNA. Data in (**B**–**D**) are means±s.e. from three independent experiments.

With the use of specific siRNAs, we next depleted EBC-1 and H1993 cells of MET, EGFR, HER2, HER3, or RET. Immunoblot analysis confirmed that transfection of cells with each siRNA resulted in the marked and selective depletion of the targeted RTK (Figure 4A). Phosphorylation of AKT and ERK was inhibited by depletion of MET, EGFR, HER2, or HER3, but not by that of RET, whereas phosphorylation of STAT3 was inhibited by depletion of MET, HER2, or RET (Figure 4A). We further examined the effects of depletion of these RTKs on cell proliferation and apoptosis in *MET* amplification-positive cells. Depletion of MET resulted in marked inhibition of cell proliferation and induced a substantial level of apoptosis in these cells, whereas depletion of EGFR, HER2, or HER3 elicited similar effects but to a lesser extent (Figure 4B and C). Depletion of RET had little effect on cell proliferation or apoptosis. Finally, we examined the effects of RTK depletion on cell migration. Depletion of MET, HER2, or RET reduced the number of migrated cells compared with that apparent for cells transfected with a control siRNA, whereas depletion of EGFR or HER3 had no substantial effect on the migration of EBC-1 or H1993 cells (Figure 4D). Together, these results suggested that MET, EGFR, HER2, and HER3 activate AKT and ERK signalling pathways and promote cell proliferation and survival in lung cancer cells with *MET* amplification, whereas MET, HER2, and RET activate the STAT3 signalling pathway and promote cell migration.

## Discussion

We have here shown that MET associates with EGFR, HER2, HER3, and RET, and that these heterodimerisation partners of MET are highly phosphorylated in lung cancer cells positive for *MET* amplification. MET was previously shown to associate with HER3 in EGFR–TKI-resistant NSCLC cells with acquired *MET* amplification (Engelman *et al*, 2007), but the regulation of such association has remained unclear. We have now found that MET also forms a complex with HER3 in lung cancer cells with endogenous amplification of *MET* and that the heterodimer was dissociated as a result of inhibition of MET kinase activity. Given that HER3 is a kinase-inactive protein and requires a dimerisation partner to become phosphorylated (Guy *et al*, 1994; Kim *et al*, 1998; Holbro *et al*, 2003), our data indicate that the kinase activity of MET is required for formation of the MET–HER3 heterodimer, which results in the phosphorylation of HER3, in lung cancer cells with *MET* amplification, although it remains unclear whether HER3 phosphorylation is mediated by MET directly or by another kinase that is activated by MET.

Dimerisation of RTKs is a key event in signal transduction (Olayioye *et al*, 2000), but much remains unknown with regard to the heterodimerisation partners of RTKs expressed on the surface of cancer cells. To identify heterodimerisation partners of MET in lung cancer cells with *MET* amplification in a comprehensive manner, we made use of an RTK array. With this approach, we identified several highly phosphorylated RTKs, including EGFR, HER2, HER3, and RET. Moreover, immunoprecipitation analysis revealed that each of these RTKs formed a heterodimer with MET, demonstrating for the first time that EGFR, HER2, HER3, and RET are the main heterodimerisation partners of MET in *MET* amplification-positive lung cancer cells. In contrast to HER3, EGFR, HER2, and RET each possess intrinsic kinase activity (Takahashi *et al*, 1988; Schlessinger, 2002). We found that the EGFR–TKI gefitinib did not inhibit EGFR phosphorylation in lung cancer cells with *MET* amplification. In addition, phosphorylation of HER2 or RET was not inhibited by the corresponding specific TKIs lapatinib or vandetanib. These results thus suggest that the observed phosphorylation of EGFR, HER2, and RET is not attributable to autophosphorylation. Moreover, we found that the MET–TKI PHA-665752 induced the dissociation of heterodimers containing MET and either EGFR, HER2, HER3, or RET, and that this effect was accompanied by dephosphorylation of these RTKs. Taken together, these data thus suggest that the association between MET and these RTKs is dependent on MET kinase activity and results in their trans-phosphorylation by MET in lung cancer cells with *MET* amplification.

**Figure 5:**
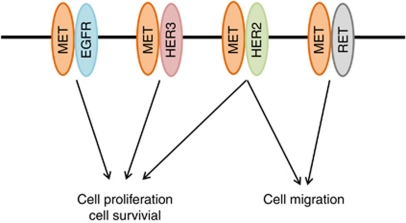
Proposed model for the roles of signalling pathways activated by heterodimers of MET and either EGFR, HER2, HER3, or RET in lung cancer cells with *MET* amplification.

We found that siRNA-mediated depletion of EGFR, HER2, or HER3 resulted in inhibition of cell proliferation and induction of apoptosis accompanied by inhibition of AKT and ERK signalling pathways in lung cancer cells with *MET* amplification, although these effects were less pronounced that were those of MET depletion. Given that these RTKs associate predominantly with MET, our data suggest that heterodimers of MET with EGFR, HER2, or HER3 activate AKT and ERK signalling and thereby promote cell proliferation and survival (Figure 5). EGFR, HER2, and HER3 appear to activate signalling by forming a complex with MET in a manner dependent on MET kinase activity rather than on their own kinase activity in lung cancer cells with *MET* amplification. We also found that heterodimers of MET with either HER2 or RET activate STAT3 signalling and likely thereby promote cell migration (Figure 5). Heterodimerisation of MET with different partners thus appears to result in different downstream effects, although the underlying mechanisms responsible for such differences remain to be elucidated. Activation of the kinase JAK by growth hormone has been shown to result in phosphorylation of EGFR and consequent activation of the ERK signalling pathway. This activation of the ERK pathway is not dependent on the kinase activity of EGFR, but is mediated by docking sites on EGFR for Grb2, consistent with the notion that RTKs may also contribute to signal transduction by functioning as adaptor proteins (Yamauchi *et al*, 1997). It is thus possible that trans-phosphorylated EGFR, HER2, HER3, and RET act as scaffold proteins to promote downstream signalling of MET in lung cancer cells with *MET* amplification. Given that inhibition of MET kinase activity resulted in inhibition of signalling pathways activated by all the heterodimers of MET with the other RTKs, MET seems to be positioned at the apex of a signalling network in *MET* amplification-positive lung cancer cells, explaining why MET inhibition is associated with pronounced antitumour effects in these cells.

We found that, whereas HER3 depletion resulted in downregulation of AKT and ERK phosphorylation, it appeared to induce upregulation of STAT3 phosphorylation in cells with *MET* amplification. Furthermore, depletion of RET inhibited STAT3 phosphorylation but appeared to increase ERK phosphorylation in such cells. Inhibition of one signalling pathway has previously been found to result in activation of other signalling pathways (Carracedo *et al*, 2008; Hoeflich *et al*, 2009; Pratilas *et al*, 2009). Such feedback regulation might explain the increases in STAT3 and ERK phosphorylation observed in this study, although further investigation is required to evaluate this notion.

In conclusion, our results suggest that EGFR, HER2, HER3, and RET are trans-phosphorylated by MET and promote cell proliferation and survival or cell migration as heterodimerisation partners of MET in lung cancer cells with *MET* amplification (Figure 5). Targeted therapeutic approaches for lung cancer, including treatment with TKIs for EGFR or the fusion protein EML4-ALK, have seen substantial progress over the last few years (Sequist *et al*, 2008; Kwak *et al*, 2010). Although amplification of *MET* is not common in NSCLC, lung tumours with *MET* amplification are highly dependent on MET signalling for their development (Lutterbach *et al*, 2007). Amplification of *MET* may thus define a subgroup of tumours that are susceptible to targeted kinase inhibition. An understanding of signal transduction in tumours with *MET* amplification will thus be important for further development of MET-targeted therapy. Our study provides new insight into the functional roles of trans-phosphorylated RTKs in *MET* amplification-positive lung cancer.
